# Investigating the “sex paradox” in pulmonary arterial hypertension: Results from the Pulmonary Hypertension Association Registry (PHAR)

**DOI:** 10.1016/j.healun.2024.02.004

**Published:** 2024-02-13

**Authors:** Jacqueline T. DesJardin, Noah Kime, Nicholas A. Kolaitis, Richard A. Kronmal, Matthew R. Lammi, Stephen C. Mathai, Corey E. Ventetuolo, Teresa De Marco

**Affiliations:** a Department of Medicine, University of California San Francisco, San Francisco, California; b Department of Biostatistics, University of Washington, Seattle, Washington; c Comprehensive Pulmonary Hypertension Center – University Medical Center, Louisiana State University, New Orleans, Louisiana; d Department of Medicine, Johns Hopkins University, Baltimore, Maryland; e Department of Medicine and Health Services, Policy and Practice, Brown University, Providence, Rhode Island

**Keywords:** pulmonary arterial hypertension, sex, hemodynamics, mortality, epidemiology

## Abstract

**BACKGROUND:**

Female sex is a significant risk factor for pulmonary arterial hypertension (PAH), yet males with PAH have worse survival – a phenomenon referred to as the “sex paradox” in PAH.

**METHODS:**

All adult PAH patients in the Pulmonary Hypertension Association Registry (PHAR) with congruent sex and gender were included. Baseline differences in demographics, hemodynamics, functional parameters, and quality of life were assessed by sex. Kaplan-Meier survival analysis was used to evaluate survival by sex. Mediation analysis was conducted with Cox proportional hazards regression by comparing the unadjusted hazard ratios for sex before and after adjustment for covariates. The plausibility of collider-stratification bias was assessed by modeling how large an unmeasured factor would have to be to generate the observed sex-based mortality differences. Subgroup analysis was performed on idiopathic and incident patients.

**RESULTS:**

Among the 1,891 patients included, 75% were female. Compared to men, women had less favorable hemodynamics, lower 6-minute walk distance, more PAH therapies, and worse functional class; however, sex-based differences were less pronounced when accounting for body surface area or expected variability by gender. On multivariate analysis, women had a 48% lower risk of death compared to men (Hazard Ratio 0.52, 95% Confidence interval 0.36 – 0.74, *p* < 0.001). Modeling found that under reasonable assumptions collider-stratification could account for sex-based differences in mortality.

**CONCLUSIONS:**

In this large registry of PAH patients new to a care center, men had worse survival than women despite having more favorable baseline characteristics. Collider-stratification bias could account for the observed greater mortality among men.

Female sex is one of the strongest known risk factors for pulmonary arterial hypertension (PAH), and yet males with PAH have worse survival – a phenomenon which has been referred to as the “sex paradox” or “sex puzzle” in PAH.^1-6^ Various reasons for the poorer survival of males have been proposed, including worse hemodynamics,^2,6-8^ less pulmonary vascular remodeling,^9^ more immune dysregulation,^10,11^ less aggressive treatment,^12^ and variable responses to therapy.^13,14^ The leading hypothesis revolves around sex-based differences in the right ventricle (RV).^6,15,16^ Under physiologic conditions, males have lower RV ejection fractions than females, and their RV function may further deteriorate in response to the increased afterload of PAH.^17,18^ RV remodeling and function in response to increased afterload is a predictor of survival among patients with PAH,^3,19^ and poorer RV adaptation in males is theorized to explain their worse survival in comparison to females.^15^

An inherent assumption in these theories is an “opposite-effects” (i.e., effect modification) hypothesis, whereby female sex switches from conferring risk to conferring benefit after the diagnosis of PAH (Figure 1a). However, alternative causal frameworks are important to consider, and use of directed acyclic graphs can help elucidate the epidemiologic mechanisms by which the “sex paradox” may occur (Figure 1). Another hypothesis would consider female sex as always harmful in PAH. In this alternative causal framework, females may appear to have lower mortality as a result of a specific form of selection bias called collider-stratification bias, which has been proposed to explain a number of epidemiologic “paradoxes” including the obesity paradox and the birthweight paradox.^20,21^ In collider-stratification bias, stratifying a diseased population by a risk factor for that disease induces a statistical association between the risk factor and the outcome, making the risk factor appear protective. This is because there are multiple causal pathways leading to the disease, of which some may be independently associated with mortality more strongly than the risk factor under examination (Figure 1c).

In this study, we sought to further investigate the “sex paradox” in PAH using data from the Pulmonary Hypertension Association Registry (PHAR), a multicenter United States-based registry of PAH patients. We investigate sex-based differences in hemodynamics, functional status, health-related quality of life, therapy, and mortality. We further investigate possible explanations for sex-based differences in mortality, examining models that consider effect modification, mediation, and collider-stratification as potential causes of the observed greater mortality in males.

## Methods

As described in prior publications,^22^ PHAR is an on-going multicenter prospective registry of patients newly evaluated at a participating Pulmonary Hypertension Care Center. PHAR was launched in 2015 and collects demographic, medical history, diagnostic, medication, quality-of-life, and outcome information on patients with PAH. Additional information on PHAR has been previously published.^23^

Inclusion criteria were a diagnosis of PAH and age > 18 years old at enrollment. Incident PAH patients were those diagnosed and started on PAH therapies within 6 months of registry entry. Patients with chronic thromboembolic pulmonary hypertension, pulmonary veno-occlusive disease, persistent pulmonary hypertension of the newborn, and congenital heart disease were excluded. Patients with missing data on PAH diagnosis (*n* = 16), sex (*n* = 14), or discordant biologic sex and gender identity (*n* = 25) were excluded. Therefore, all patients included had the same sex and gender and so the terms male/man and female/woman are used interchangeably.

Standard hemodynamic variables were collected from patients’ baseline right heart catheterization. Derived hemodynamic variables, including pulmonary vascular resistance (PVR), pulmonary arterial compliance (PAC), and RV stroke work index (RVSWI) were calculated using previously described equations.^22^ PAC index was calculated by dividing the PAC by the body surface area (BSA).^24^ Analysis of 6-minute walk distance (6MWD) was performed on both the absolute (in meters) and percent predicted scale. Percent predicted 6MWD was calculated from reference equations which adjust for age, sex, height, and weight.^25^ Baseline risk was assessed using the REVEAL Lite 2 calculator, which excludes non-modifiable variables (age, sex, etiology).^26^ Health-Related Quality of Life (HRQOL) was quantified by (1) the PAH-specific instrument, Emphasis-10 (higher scores denoting worse HRQOL); (2) the generic-physical instrument, SF12-Physical; and (3) the generic-mental instrument, SF12-Mental (higher scores denoting better HRQOL). Chi-squared test, Student’s *t*-test, and Wilcoxon sign-rank test were used, as indicated, to assess for sex-based differences in baseline demographics, hemodynamics, therapies, and functional parameters.

For analysis of survival, only patients with follow-up data were included (*n* = 1,615). Kaplan-Meier survival analysis was used to compare survival time from enrollment by sex and by sex and age. Age groups (< 60 vs > 60) were chosen *a prior* to allow for comparison to REVEAL, another large US-based PAH registry.^2^ Data on sex, age, primary PH diagnosis, and vital status at follow-up were available for all patients; < 5% of data on other covariates was missing (Supplemental Table 3).

Univariate Cox proportional-hazards regression was employed to examine sex-based differences in all-cause mortality. After examining baseline differences by sex, mediation analysis was conducted to further explore the cause of sex-based differences in mortality. Models were developed to examine the relationship between sex and death while controlling for variables hypothesized to mediate the relationship. Based on the observed baseline differences by sex and previously proposed hypotheses, the following models were examined: (1) demographic differences (age and PAH diagnosis), (2) comorbidities (body mass index [BMI] and estimated glomerular filtration [eGFR]), (3) health behaviors (prior history of cigarette smoking or methamphetamine use), (4) social determinates of health (insurance, education, income, occupation), (5) 6MWD, (6) PAH therapy (number of therapies, intravenous prostacyclin therapy), and (7) hemodynamics (mean pulmonary artery pressure [mPA], pulmonary vascular resistance [PVR], cardiac index [CI], pulmonary artery wedge pressure [PAWP]). Model 3 was included because at baseline males were more likely than females to report a history of tobacco or methamphetamine use (Table 1), and these behaviors are independently associated with mortality.^27,28^ Model 4 was included because social determinants of health were found to vary by sex at baseline (Supplemental Table 1) and may account for differences in mortality. All other models were chosen because the included variables have been associated with mortality in PAH and/or have been previously proposed to mediate sex-based differences in mortality. Mediation analysis was conducted by using univariate and multivariate Cox regression and comparing the unadjusted log hazard ratios for sex before and after additional adjustment for covariates. The fully-adjusted multivariate model adjusted for all variables: age, PAH diagnosis, BMI, eGFR, methamphetamine use, smoking, income, education, occupation, insurance, 6MWD, number of PAH therapies, intravenous prostacyclin therapy, mPA, PVR, CI, and PAWP. Number of PAH therapies and intravenous prostacyclin therapy were analyzed as time-dependent covariates thereby accounting for changes in PAH therapy overtime during follow up; all other covariates were recorded at baseline.

Recent advancements in epidemiology have allowed for modeling of collider stratification bias, which can be used to assess the magnitude of the potential bias in a given population.^29,30^ Using previously published methods, we modeled how large an unmeasured factor (U) would have to be to generate mortality differences by sex via collider-stratification.^30^ The goal of this analysis is to understand the epidemiologic conditions under which collider-stratification could plausibly account for the sex-based mortality differences in PAH. If, under reasonable assumptions, modeling of collider-stratification bias recapitulates the lower mortality in women, one could conclude that collider-stratification may account for the observed “sex paradox” in PAH. Using the input assumptions specified in the footnote of Table 3 and outlined in Figure 5, we calculated the risk ratios (RR) for the effect of female sex on mortality among PAH patients in an analysis not adjusting for U. In other words, we assume there is an unmeasured factor (U) causing collider-stratification and then we look to see what assumptions we would have to make around U in order to get modeled risk ratios which align with the observed sex-paradox phenomenon. The hypothetical risk ratios (RR) for mortality by sex obtained under modeling conditions were then compared to the actual RR obtained from PHAR to assess the conditions under which collider-stratification bias could account for the observed greater mortality in men.

Pre-specified subgroup analysis was performed on idiopathic and incident patients. In order to assess if missing data substantially influenced the results, sensitivity analysis was performed using multiple imputation of missing data (Supplemental Figure 1). Statistical analysis was performed with STATA version 16 and R. *P* < 0.05 was considered statistically significant.

## Results

Among the 1,891 PAH patients included, men had more drug/toxin-associated and portopulmonary PAH, while women had more connective tissue disease-associated PAH. Age, race, ethnicity, BMI, and eGFR were comparable between sexes (Table 1). Men and women had similar incomes, but varied substantially in their employment, smoking, and methamphetamine use histories, with men having higher risk behaviors (Supplemental Table 1). At baseline, women were more likely to be on triple therapy or parenteral prostacyclin therapies (Table 2). Men had higher PAWPs than women (Table 2). Compared to men, women had worse baseline hemodynamics including higher PVR, lower PAC, and lower CO. However, women had significantly smaller body surface areas (BSA) relative to men, and there were no differences in the indexed versions of these hemodynamic variables (e.g., PVRi, PACi, CI) (Table 2). Unlike the other hemodynamic variables, RVSWI (which incorporates BSA), was significantly lower in women compared to men. Women had higher risk and worse functional status as measured by the REVEAL Lite 2 Risk score, World Health Organization (WHO) functional class, emPHasis-10 score, and 6MWD. The percent predicted 6MWD did not differ by sex (Table 2).

Among 1,615 patients (387 men; 1,228 women) with follow-up data available, there were 307 deaths (92 in men, 215 in women) over a median follow up of 23 months (IQR 12–41). In Kaplan-Meir survival analysis, men had significantly poorer survival compared to women (log-rank test *p* = 0.006, Figures 2 and 3a), and survival was especially poor among men over 60 years of age (Figure 3b). In univariate cox regression, women had a 29% lower risk of death compared to men (HR 0.71, 95% CI 0.56–0.91, *p* = 0.007). In the fully-adjusted model, women had a 48% lower risk of death compared to men (HR 0.52, 95% CI 0.36–0.74, *p* < 0.001). On mediation analysis, differences in demographics (age, PAH diagnosis), comorbidities (BMI, eGFR), health behaviors (smoking, methamphetamine use), social determinants of health (income, occupation, education, insurance), 6MWD, change in PAH therapy over time (none vs mono vs dual vs triple; intravenous prostacyclin therapy), and hemodynamics (mPA, PVR, CI, PAWP) did not fully account for the mortality difference by sex (Figure 4).

In modeling if a further unmeasured factor (U) could plausibly account for the greater mortality among men via collider-stratification bias, we found that under the input assumptions specified in the footnote of Table 3, unadjusted RRs of < 1.0 were obtained for the effect of female sex on mortality among patients with PAH (Table 3). Many of the modeled RR were either within (Table 3, green) or below (Table 3, red) the 95% confidence interval of the observed data, meaning modeling of collider-stratification bias recapitulated the observed sex paradox. For example, assuming (1) a RR for the association of U with PAH in men of 2.0, (2) a prevalence of U in the population without PAH of 0.05, (3) a RR for the association of female sex with mortality of 1.0, and (4) a RR for the association of U with mortality of 5.0 would generate an unadjusted RR of 0.87. When female sex is itself considered a protective factor against death in the general population, the modeled unadjusted RRs for the effect of female sex on mortality among PAH patients are < 0.50 (Table 3). Additional modeling conditions are included in Supplemental Table 4.

Subgroup analysis in incident (*n* = 998) and idiopathic (*n* = 817) patients found comparable results to the full analysis (Supplemental Table 2). Sensitivity analysis using multiple imputation to account for missing data produced comparable results: as expected, the precision of hazard ratio estimates was improved with multiple imputation methods (Supplemental Figure 1). In the fully-adjusted model with multiple imputation, women had a 36% lower risk of death compared to men (HR 0.64, 95% CI 0.60–0.69, *p* < 0.001).

## Discussion

In this analysis of a large multicenter cohort of PAH patients, female sex was once more shown to be a significant risk factor for PAH. At baseline, women appeared to have more severe disease in terms of functional status, risk scores, HRQOL, and non-indexed hemodynamic variables. And yet, female sex was again protective against death among PAH patients in PHAR. Despite examination of a number of potential mediating factors, none of the variables collected in PHAR and examined in this study fully explained the increased mortality in men. Modeling demonstrated that an unmeasured factor or factors could plausibly account for the sex-based differences in mortality via collider-stratification bias.

**Figure 1:**
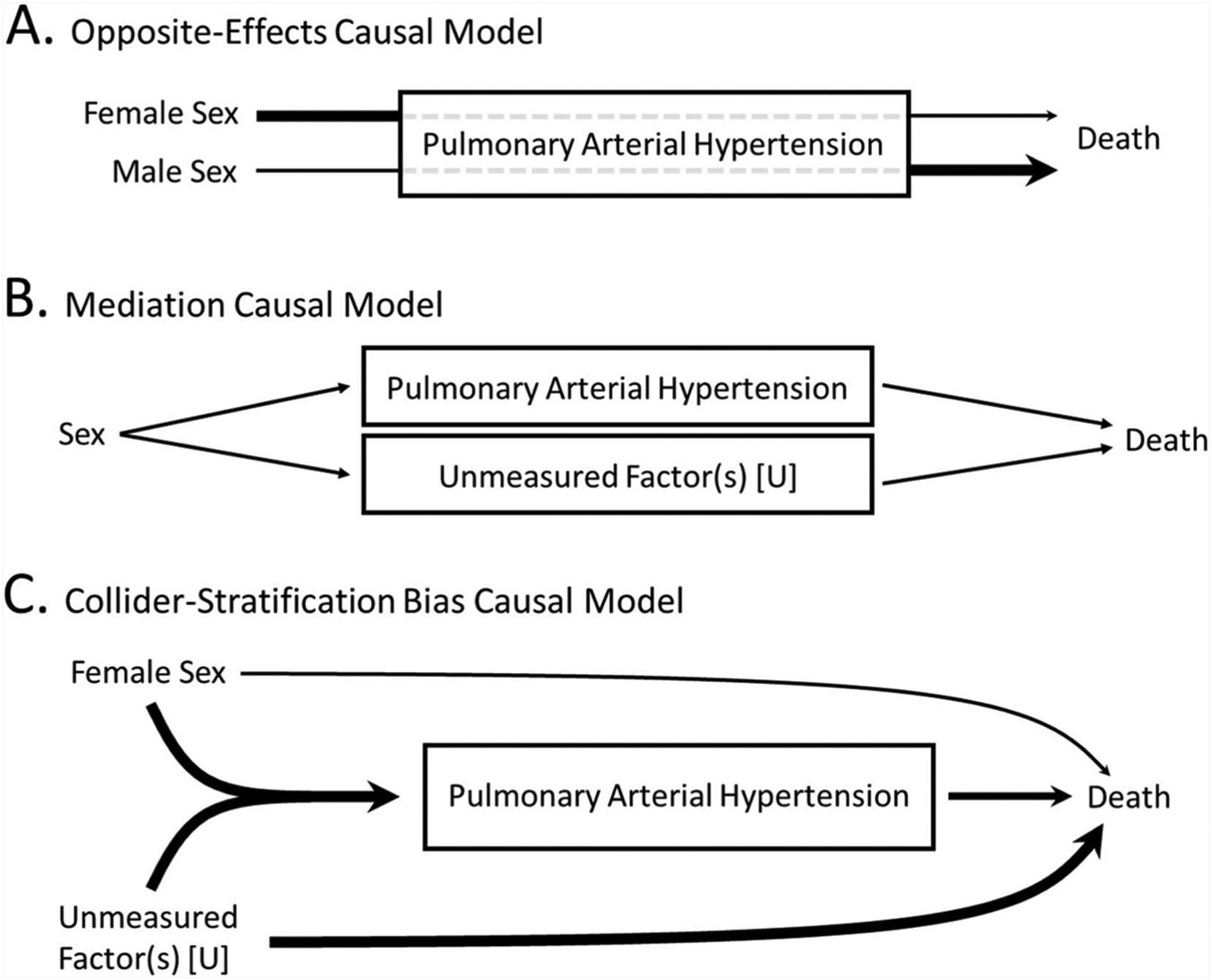
Potential causal models explaining the sex paradox in pulmonary arterial hypertension.

**Figure 2:**
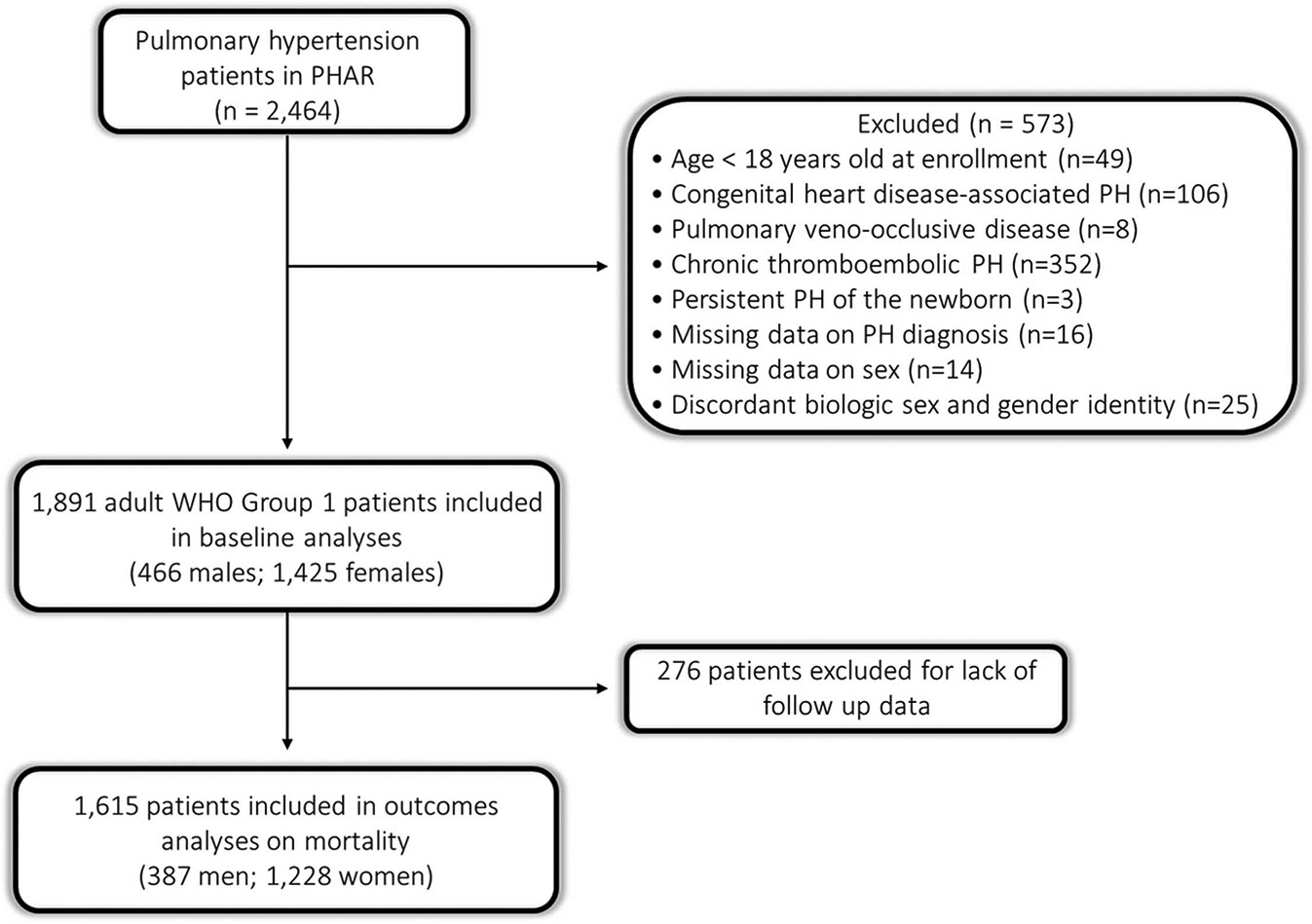
Consort diagram.

**Figure 3:**
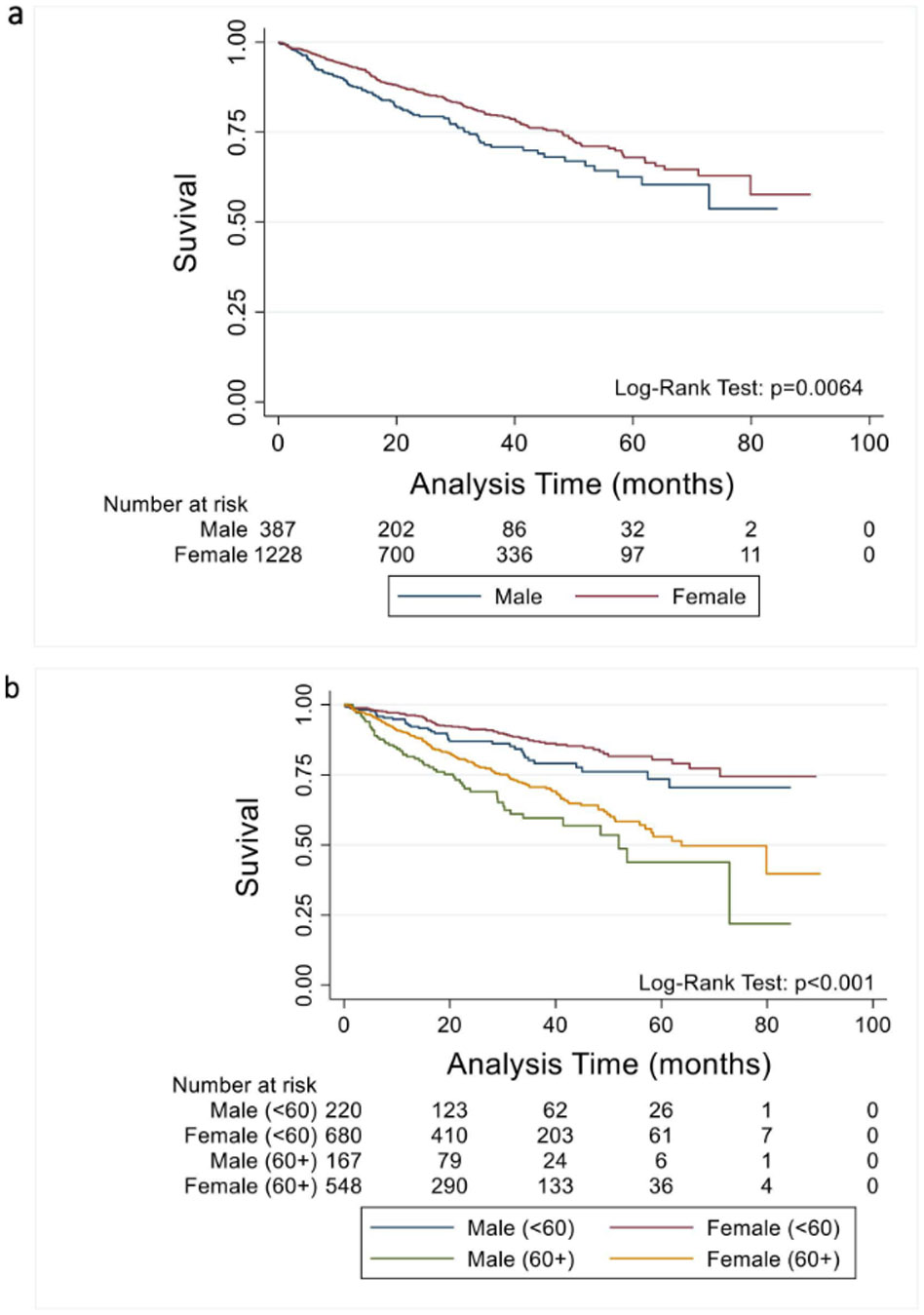
Kaplan Meier survival analysis. (a) Kaplan Meier survival analysis by sex. (b) Kaplan Meier survival analysis by sex and age.

**Figure 4:**
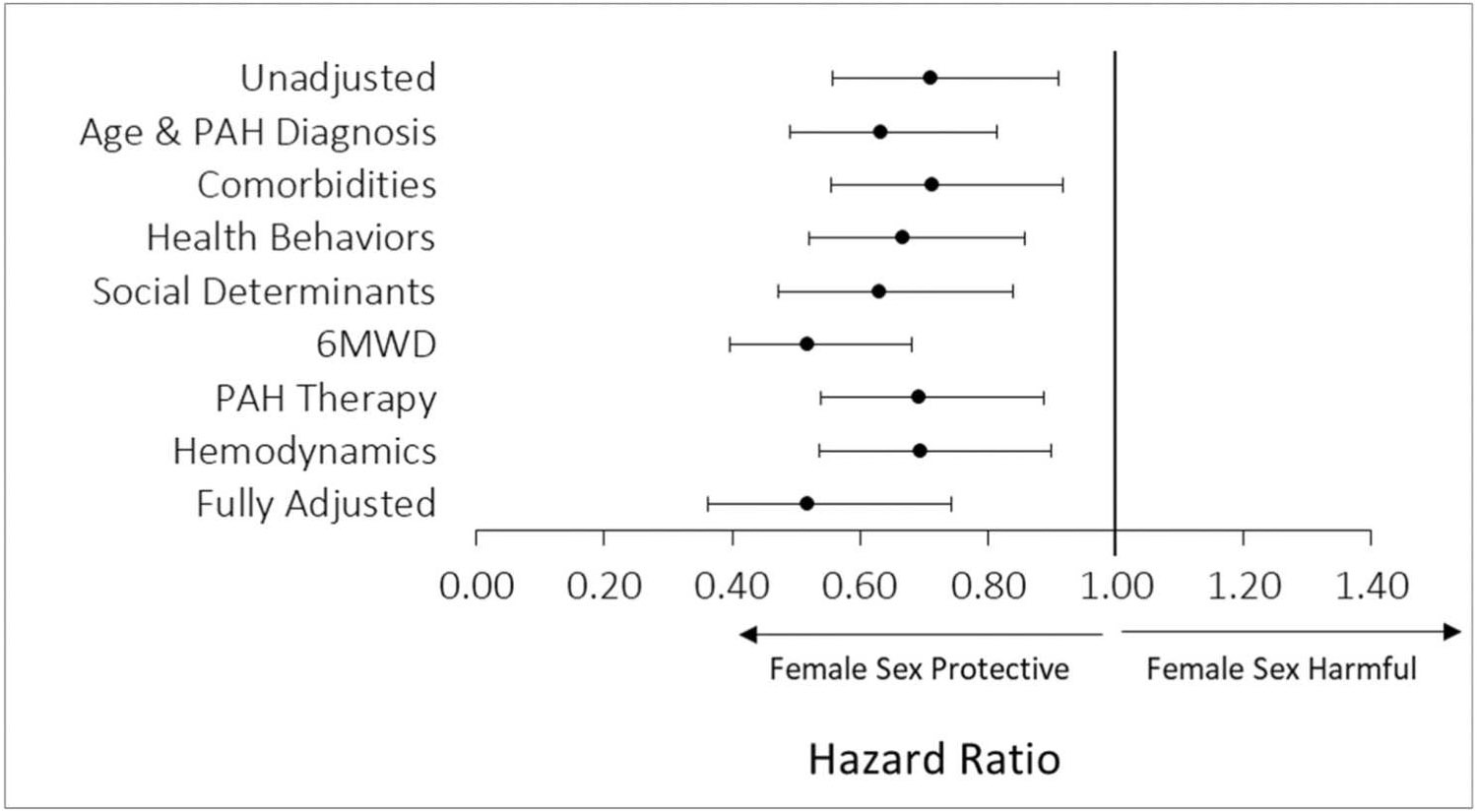
Mediation analysis examining the hazard ratio and 95% confidence intervals for unadjusted and adjusted models of the association between sex and mortality.

**Figure 5:**
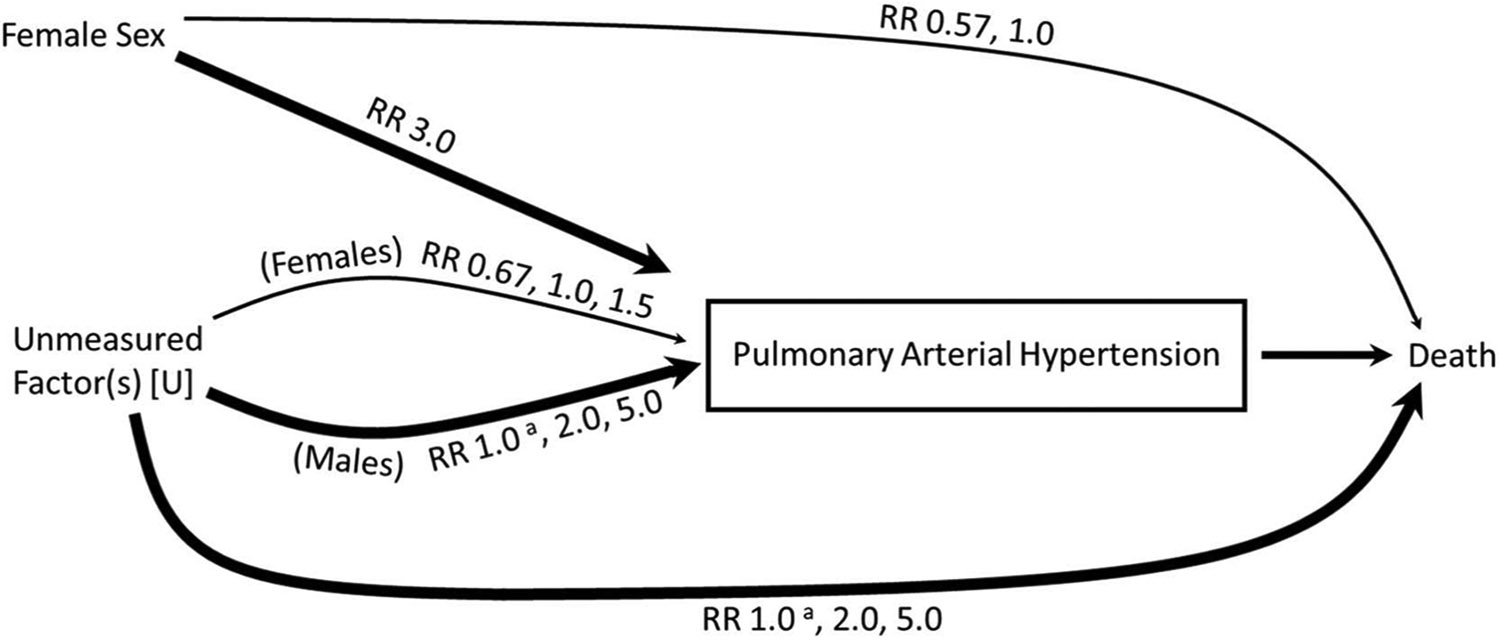
Risk Ratio assumptions used for modeling of collider-stratification bias in Table 3. ^a^ = Modeling is included in the supplemental materials; RR, risk ratio.

Many theories have been proposed to explain the sex paradox in PAH. These theories can largely be categorized into 3 theoretical causal models: (1) an opposite-effects (effect modification) model, (2) a mediation model, and (3) a collider-stratification model (Figure 1). Notably, these models are not mutually exclusive: either model, or a combination of them, could account for the consistently observed greater mortality among men. The purpose of this study was to explore the plausibility of each causal models in the context of prior research on the sex paradox in PAH.

Consistent with data from prior registries, we found that female sex is a significant risk factor for PAH, with women accounting for 75% of PAH patients in PHAR, even in this registry of largely incident (within 6 months of referral) patients. Prior registries have similarly shown a high prevalence in women, with women comprising anywhere between 56% (Swedish PAH Registry [SPAHR]) and 83% (REVEAL) of registry participants.^5,31^ The female predominance in PAH declines with age, and in the European-based registry COMPERA the female-to-male ratio was nearly even among patients over 65 years old.^32^ As in REVEAL and SPAHR, we showed that sex-based survival differences in PHAR were greatest in older PAH patients, re-emphasizing an important interaction between sex, age, and mortality among PAH patients.^2,3^

In concordance with an opposite-effects causal model are theories which hypothesize that sex and its associated hormonal differences are responsible for the greater mortality in men. In this theoretical framework, while women are at higher risk of pulmonary vascular remodeling and PAH, men have maladaptive RV responses to the hemodynamic stressors induced by PAH.^33^ In PHAR and in prior studies, baseline invasive hemodynamics have largely been similar between sexes, especially when controlling for differences in body size. However, some studies have suggested that women have better RV function and response to PAH therapies. In a study of 101 PAH patients at a single center in The Netherlands, men had larger baseline RV volume indexes on cardiac magnetic resonance imaging (CMR) despite similar invasive hemodynamics. In follow-up, men had greater mortality which was attributable to larger deteriorations of RV function (as measured by RV ejection fraction on CMR) after initiation of therapy.^15^ Sex differences in response to therapy have been previously described in PAH, albeit with somewhat conflicting findings. Women have been reported to respond more favorably to endothelin receptor antagonists (ERA) but less favorably to phosphodiesterase 5 inhibitors. Pooled analyses including 1,130 patients from 6 placebo-controlled trials of ERAs found that women had greater improvement in 6MWD with ERA treatment relative to men.^13^ In post-hoc analysis of the PHIRST trial (tadalafil versus placebo), women treated with tadalafil had a lower odds of achieving significant improvement in the 6MWD or SF-36,^14^ with some suggestion that postmenopausal women derive the least benefit.^34^ In PHAR, differences in PAH therapy over time did not fully explain sex-based differences in mortality. Our Cox regression analysis controlled for change in number of therapies as well as intravenous prostacyclin therapy over time, but did not control for specific drug classes nor did we control for medication adherence, which may have varied by sex.

Sex is known to influence RV size and function. In comparison to women, men without known cardiovascular disease have larger RV masses and volumes, yet 4% lower RV ejection fractions on cardiac MRI.^18^ In PAH, female patients have been shown to have better RV-PA coupling, diastolic RV relaxation, and lower RV mass index in comparison to male PAH patients.^35^ Sex hormones are thought to mediate sex-based differences in the RV and its response to increased afterload. Compared to controls, menstruating women with PAH have lower dehydroepiandrosterone sulfate (DHEA-S) levels but higher and less variable estradiol levels, with levels of both hormones predictive of 6MWD and natriuretic peptides.^36^ In the Multi-Ethnic Study of Atherosclerosis, higher estradiol levels were associated with better RV systolic function in post-menopausal women on hormone replacement, while androgens were associated with larger RV masses and volumes.^37^ In models of RV pressure overload by pulmonary trunk binding, male rodents have demonstrated greater RV hypertrophy and natriuretic peptide release relative to females, and testosterone has been shown to induce more RV fibrosis, increased myocyte size, and worsened survival.^38,39^ PHAR does not include data on CMR or echocardiography, and only 6 patients have had follow up hemodynamics to date, which precludes analysis on change in hemodynamic variables over time. Therefore, our ability to test the hypothesis that greater RV failure over time contributes greater mortality in men is limited.

An alternative explanation for sex-based differences in mortality, which is not necessarily mutually exclusive with opposite-effects framework, would be to consider an external and unmeasured factor or factors as leading to the sex paradox in PAH. Recently methodologic advances have allowed for collider-stratification bias to be recognized as the underlying cause of many long-standing “paradoxical” findings in epidemiology, including the obesity paradox^21^ and the birthweight paradox.^20^ The classical example is the “birthweight paradox” — among infants with low birthweight, those with mothers who smoked had a lower mortality compared to those with mothers who did not smoke. For many years it was postulated that smoking somehow reduced the risk of death in low birthweight infants. However, it is now understood that the birthweight paradox was a consequence of collider-stratification bias.^20^ In simplified terms, there are multiple pathways leading to development of a low birthweight infant, of which smoking is only one. The other causal pathways leading to low birthweight (i.e., genetic abnormalities in the infant) are themselves strongly associated with mortality. Therefore, when the population is conditioned based on disease (low birthweight) and then stratified on a risk factor for that disease (smoking), a statistical association is induced such that the risk factor (smoking) appears falsely protective. A similar effect may occur with PAH. In the case of PAH, men with PAH do die more than women, but this may be because men require a higher-risk causal pathway to get PAH which is itself independently associated with death, not because they are men per se. The so-called “sex paradox” in PAH would therefore not be a paradox whatsoever, but rather an expected consequence of a well-described epidemiologic principle. In modeling the collider-stratification bias, we found that it could plausibly account for sex-based differences in mortality under reasonable input assumptions. This is different from similar modeling exercises in other populations, in which collider-stratification bias was found to be possible but not probable because the unmeasured factor(s) would have to exert such a large effect that it would be biologic implausible.^30^

While the unmeasured factor or factors accounting for collider-stratification remain unclear, modeling demonstrates that they may be relatively small in prevalence and effect magnitude yet still substantially bias statistical analyses. Furthermore, collider-stratification is reasonable in this epidemiologic context, as available data do suggest that men may require a higher-risk causal pathway to get PAH. In PHAR, men had higher risk behaviors and higher risk causes of PAH (i.e., portopulmonary). Although we are controlling for these factors, residual risk may have been unaccounted for and higher risk behaviors may in fact be the unmeasured factors which contribute to greater mortality in men. Take for example methamphetamine-associated PAH: a PAH subtype more likely to affect men and also associated with high mortality.^40^ In a case-control study of people with methamphetamine use, women with methamphetamine use are more likely to develop PAH while men are more likely to develop dilated left-ventricular cardiomyopathies.^41^ The small percentage of men who do develop PAH as a consequence of methamphetamine use likely require additional “hits” predisposing to PAH (i.e., double-hit hypothesis). These additional hits might include HIV, cirrhosis, genetic predisposition, or longer exposure to methamphetamines – all of which may themselves be associated with higher mortality. The hypothesis that individuals require a double-hit to develop PAH is supported by the findings that illicit drug use increases the risk of HIV-associated PAH, and that *BMPR2* mutations increase the risk of fenfluramine-associated PAH.^42,43^ Men who develop PAH may therefore require several higher-risk hits (e.g., HIV and methamphetamine use), while women who develop PAH may get the disease more easily via causal mechanisms that are not as strongly associated with mortality. These causal pathways leading into the diagnosis of PAH are likely variable and our current registries are not well-designed to explore or control for them. Contemporary PAH registries primarily focus on management following PAH diagnosis rather than looking back at the pathway leading into the PAH diagnosis. Risk factors for PAH are quantified in binary terms (i.e., HIV yes/no, methamphetamine use yes/no) rather than further measuring their severity, and PAH etiology is classified into mutually-exclusive groups rather than allowing for multiple or overlapping phenotypes. More nuance is required to adequately control for these causal pathways and account for collider-stratification bias in registry data. Researchers must understand how causal factors which exist and are unmeasured prior to PAH diagnosis can affect their statistical analyses and the resulting conclusions.

Unlike other PAH registries, PHAR collects detailed data on both biologic sex, gender identity, demographics, and social determinates, allowing for a more nuanced analysis of these factors. Nonetheless, this study is limited by the inherent biases (i.e., selection bias), data quality confines, and unavailable or missing data which is inevitable in registry-based observational research. PHAR is a recently established registry, and the available follow up for analysis was therefore shorter than in many other large PAH registries. Data from PHAR therefore reflect contemporary clinical practice in the United States; however, additional studies from PHAR after longer follow-up time has accrued will be important for confirming the reported findings. Additionally, PHAR does not collect detailed information on medical comorbidities, although variation in comorbidities by sex have been reported in PAH patients,^2^ especially among those over 65 years of age.^5^ It is possible the sex-based differences in comorbidities might account for differences in mortality via mediation. As already discussed, PHAR does not collect data on echocardiography, CMR, and several important comorbidities, and data on follow-up hemodynamic assessments are limited. Additionally, the diffusing capacity of the lungs for carbon monoxide (DLCO) has been established as an important prognostic indicator in PAH but is not collected in PHAR. We are unable to examine to what extent sex-differences in DLCO may account for sex-differences in outcomes. These limitations prevent a fully comprehensive investigation of the causes of sex-based differences in mortality.

Ultimately, the cause of greater mortality in men remains unclear, and may be multifactorial. Adverse hormonally-based RV responses to afterload and therapy in men and/or unmeasured factor(s) such as comorbidities may contribute to the observed sex-based differences in mortality. While sex may be an immutable factor, a better understanding of the causal structure underlying the “sex paradox” in PAH provides foundations for future research and informs investigations of hormonal-based therapies for PAH. In the future, registries should emphasize collecting more detailed data on PAH risk factors and avoid the over-simplification of classification of PAH etiologies. In this paper, we sought to organize theories around sex, gender, and PAH into a clearly defined causal framework in hopes of facilitating further research on the topic. We hope the paper will shed light on how epidemiologic principles may bias statistical inferences in registry-based studies.

## Supplementary Material

supplemental materials

## Figures and Tables

**Table 1:** Baseline Characteristics of Adult PAH Patients Enrolled in PHAR by Sex

	Total	Male	Female	*p*-value
N	1,891	466	1,425	-
Age, Mean (SD)^a^	56 (16)	56 (15)	56 (16)	0.75
Race, *n* (%N)^b^				
White	1,427 (75%)	371 (80%)	1,056 (74%)	0.10
Black or African American	240 (13%)	41 (9%)	199 (14%)	
Asian	71 (4%)	16 (3%)	55 (4%)	
American Indian or Alaskan Native	17 (1%)	4 (1%)	13 (1%)	
Native Hawaiian or Other Pacific Islander	6 (< 1%)	2 (< 1%)	4 (< 1%)	
More than 1 race or unknown	130 (7%)	32 (7%)	98 (7%)	
Ethnicity, *n* (%N)^b^				
Non-Hispanic or Latino	1,608 (85%)	400 (86%)	1,208 (85%)	0.69
Hispanic or Latino	194 (10%)	43 (9%)	151 (11%)	
Unknown or missing	89 (5%)	23 (5%)	66 (5%)	
PAH Diagnosis, *n* (%N)^b^				
Idiopathic	817 (43%)	193 (41%)	624 (44%)	< 0.01
Connective tissue disease-associated	617 (33%)	85 (18%)	532 (37%)	
Drug/toxin-associated	243 (13%)	91 (20%)	152 (11%)	
Portopulmonary	127 (7%)	60 (13%)	67 (5%)	
Heritable	55 (3%)	14 (3%)	41 (3%)	
HIV-related	32 (2%)	23 (5%)	9 (1%)	
Comorbidities, Mean (SD)^a^				
Body mass index, kg/m^2^ (*n* = 1,847)	30 (7)	30 (6)	30 (8)	0.41
eGFR, mL/min/1.73 m^2^ (*n* = 1,838)	78 (26)	79 (26)	77 (26)	0.34

eGFR, estimated glomerular filtration rate, as calculated by the Modification of Diet in Renal Disease [MDRD] equation; HIV, human immunodeficiency virus; PAH, pulmonary arterial hypertension; PHAR, Pulmonary Hypertension Association Registry.

a Student’s t-test.

b Chi-squared test.

**Table 2:** Baseline Hemodynamics, Therapies, and Functional Parameters by Sex

	Total (N = 1,891)	Male (N = 466)	Female (N = 1,425)	*p*-value
Hemodynamics, Median (IQR)^a^				
Right atrial pressure, mm Hg (*n* = 1,798)	9 (5, 14)	9 (6, 13)	9 (5, 14)	0.24
Mean pulmonary artery pressure, mmHg (*n* = 1,838)	48 (39, 57)	48 (40, 58)	49 (39, 57)	0.20
Pulmonary artery wedge pressure, mmHg (*n* = 1,816)	10 (7, 14)	11 (8, 14)	10 (7, 14)	**< 0.01**
Pulmonary vascular resistance, WU (*n* = 1,712)	9 (6, 13)	8 (6, 12)	9 (6, 14)	**< 0.01**
Pulmonary artery compliance, ml/mm Hg (*n* = 1,280)	1.2 (0.8, 1.7)	1.3 (1.0, 1.8)	1.1 (0.8, 1.6)	**< 0.01**
Cardiac output, L/min (*n* = 1,756)	4.0 (3.2, 5.1)	4.5 (3.6, 5.6)	3.9 (3.1, 5.0)	**< 0.01**
Body surface area, m^2^ (*n* = 1,847)	1.9 (1.7, 2.1)	2.1 (1.9, 2.2)	1.8 (1.7, 2.0)	**< 0.01**
Pulmonary vascular resistance index, WU*m^2^ (*n* = 1,678)	17 (12, 24)	17 (12, 24)	17 (11, 24)	0.46
Pulmonary artery compliance index, ml/mm Hg/m^2^ (*n* = 1,251)	0.6 (0.4, 0.9)	0.6 (0.5, 0.9)	0.6 (0.4, 0.9)	0.15
Cardiac index, L/min/m^2^ (*n* = 1,720)	2.2 (1.7, 2.7)	2.2 (1.8, 2.6)	2.1 (1.7, 2.7)	0.99
RV stroke work index, g*m/m^2^/beat (*n* = 1,235)	14 (11, 18)	15 (11, 19)	14 (10, 18)	**< 0.01**
PAH Therapies, *n* (%N) (*n* = 1,889)^b^				
Number of PAH Therapies				
0 – No PAH-specific therapies	211 (11%)	55 (12%)	156 (11%)	**< 0.01**
1 – Mono therapy	455 (24%)	125 (27%)	330 (23%)	
2 – Dual therapy	852 (45%)	224 (48%)	628 (44%)	
3 – Triple or greater therapy	371 (20%)	62 (13%)	309 (22%)	
Types of PAH Therapies				
Oral PDE5i or sGC Stimulators	1,523 (81%)	377 (81%)	1,146 (81%)	0.86
Oral Endothelin Receptor Antagonist	1,073 (57%)	252 (54%)	821 (58%)	0.17
Oral Prostacyclin Analogs	173 (9%)	38 (8%)	135 (9%)	0.39
Inhaled Prostacyclin	131 (7%)	23 (5%)	108 (8%)	0.05
Intravenous Prostacyclin	460 (24%)	82 (18%)	378 (27%)	**< 0.01**
Functional Parameters				
REVEAL Lite 2 Risk Score, *n* (%N) (*n* = 914)^b^				
Low Risk (< 5)	459 (50%)	124 (54%)	335 (49%)	0.29
Intermediate Risk (6-7)	240 (26%)	56 (25%)	184 (27%)	
High Risk (> 8)	215 (24%)	47 (21%)	168 (24%)	
WHO-FC III or IV, *n* (%N) (*n* = 1,790)^b^	997 (56%)	217 (50%)	780 (58%)	**< 0.01**
6MWD, meters, Mean (SD) (*n* = 1,700)^c^	327 (127)	357 (129)	319 (126)	**< 0.01**
% Predicted 6MWD, %, Mean (SD) (*n* = 1,667)^c^	62 (22)	61 (22)	63 (23)	0.27
emPHasis-10, Median (IQR) (*n* = 1,862)^a^	27 (16, 35)	23 (13, 32)	28 (18, 36)	**< 0.01**
SF12-Physical, Median (IQR) (*n* = 1,862)^a^	34 (27, 42)	36 (28, 43)	34 (27, 41)	**< 0.01**
SF12-Mental, Median (IQR) (*n* = 1,862)^a^	48 (39, 57)	49 (41, 58)	47 (38, 56)	**< 0.01**

PAH, pulmonary arterial hypertension; REVEAL, Registry to Evaluate Early and Long-term PAH Disease Management; WHO, World Health Organization.

Health-Related Quality of Life (HRQOL) quantified by (1) the PAH-specific instrument emPHasis-10 (higher scores denoting worse HRQOL); (2) the generic-physical instrument, SF12-Physical (higher scores denoting better HRQOL); and (3) the generic-mental instrument, SF12-Mental (higher scores denoting better HRQOL). Oral PDE5i (phosphodieseterase-5 inhibitors) and sGC (soluble guanylate cyclase) stimulators = sildenafil, tadalafil, or riociguat. Oral Endothelin Receptor Antagonist = ambrisentan, bosentan, or macitentan. Oral prostacyclin analogs = oral treprostinil or selexipag. Inhaled prostacyclin = inhaled treprostinil or inhaled iloprost. Intravenous prostacyclin = intravenous epoprostenol or treprostinil.

Bold values indicates statistically significant to degree of < 0.05.

a Rank sum test.

b Chi-squared test.

c Student’s t-test.

**Table 3:** Modeling of the Conditions Under which an Unmeasured Factor (U) May Account for Observed Sex-Based Differences in Mortality via Collider-Stratification Bias

		M.U	PAH.U	RR for association of U with PAH in men: 2						RR for association of U with PAH in men: 5					
				Prevalence of U in population without PAH						Prevalence of U in population without PAH					
**RR for association of female sex with mortality**	**1.0**			**0.05**	**0.10**	**0.20**	**0.30**	**0.40**	**0.50**	**0.05**	**0.10**	**0.20**	**0.30**	**0.40**	**0.50**
		**2**	**0.67**	0.94	0.90	0.86	0.84	0.83	0.84	0.86	0.79	0.74	0.73	0.74	0.76
		**5**	**0.67**	0.82	0.74	0.67	0.66	0.68	0.71	0.62	0.53	0.49	0.51	0.55	0.60
		**2**	**1.00**	0.96	0.93	0.90	0.89	0.89	0.90	0.87	0.81	0.77	0.77	0.79	0.82
		**5**	**1.00**	0.87	0.81	0.77	0.77	0.79	0.82	0.65	0.58	0.56	0.59	0.64	0.69
		**2**	**1.50**	0.98	0.97	0.95	0.95	0.95	0.96	0.89	0.84	0.82	0.83	0.85	0.87
		**5**	**1.50**	0.94	0.91	0.90	0.90	0.91	0.93	0.71	0.65	0.65	0.69	0.74	0.78
	**0.57**	**2**	**0.67**	0.54	0.52	0.49	0.48	0.47	0.48	0.49	0.45	0.42	0.41	0.42	0.44
		**5**	**0.67**	0.47	0.42	0.38	0.38	0.39	0.40	0.35	0.30	0.28	0.29	0.31	0.34
		**2**	**1.00**	0.55	0.53	0.51	0.51	0.51	0.51	0.50	0.46	0.44	0.44	0.45	0.47
		**5**	**1.00**	0.50	0.46	0.44	0.44	0.45	0.47	0.37	0.33	0.32	0.34	0.36	0.39
		**2**	**1.50**	0.56	0.55	0.54	0.54	0.54	0.55	0.51	0.48	0.47	0.47	0.48	0.50
		**5**	**1.50**	0.53	0.52	0.51	0.51	0.52	0.53	0.40	0.37	0.37	0.39	0.42	0.45
Key															
**RR _Model_ < RR _Observed_** Modeling estimates female sex is more protective than the observed data demonstrate (RR _Model_ < 0.60; below the lower limit of the 95% confidence interval of observed data)					**RR _Model_ = RR _observed_** Modeling estimates female sex is as protective as the observed data demonstrate (RR _Model_ 0.60 – 0.92; within the 95% confidence interval of observed data)						**RR _Model_ > RR _Observed_** Modeling estimates female sex is less protective than the observed data demonstrate (RR _Model_ > 0.92; above the upper limit of the 95% confidence interval of observed data)				

M.U. = risk ratio for association of U with mortality; PAH.U = risk ratio for association of U with PAH among women. Assumptions: (1) prevalence of PAH among men = 5 cases per million^44^; (2) Risk ratio (RR) for effect of female sex on PAH = 3.0 (as seen in PHAR); (3) mortality of men versus women in the general population = RR 1.0 (equivalent) or 0.57 (lower mortality for women, based on U.S.-based longitudinal cohorts)^45^; (4) RR for the association of U with mortality = varied between 2.0 and 5.0; (5) RR for the association of U with PAH among women = varied between 0.67 and 1.50; (6) prevalence of U in the population without PAH = varied between 0.05 and 0.50. Table is color-coded according to whether modeling of collider-stratification bias predicted RR which were below (red), equal to (green), or above (blue) the 95% confidence interval for the observed unadjusted RR for mortality by sex in PHAR (RR 0.74; 95% CI 0.60–0.92).

## Abbreviations:

6MWD: 6-minute walk distance
BMI: body mass index
BSA: body surface area
CI: cardiac index
CMR: cardiac magnetic resonance imaging
DLCO: diffusing capacity of the lungs for carbon monoxide
eGFR: estimated glomerular filtration rate
ERA: endothelin receptor antagonists
HR: Hazard Ratio
HRQOL: health-related quality of life
mPA: mean pulmonary artery pressure
PAC: pulmonary arterial compliance
PACi: pulmonary arterial compliance index
PAH: pulmonary arterial hypertension
PAWP: pulmonary artery wedge pressure
PHAR: Pulmonary Hypertension Association Registry
PVR: pulmonary vascular resistance
PVRi: pulmonary vascular resistance index
REVEAL: Registry to Evaluate Early and Long-term PAH Disease Management
RR: risk ratio
RV: right ventricular
RVSWI: right ventricular stroke work index
WHO: World Health Organization
