# Supplementary material for: Investigating the “sex paradox” in pulmonary arterial hypertension: Results from the Pulmonary Hypertension Association Registry (PHAR)
Source: J Heart Lung Transplant. Author manuscript; Available in PMC 2024 Oct 24. (PMC11500812; doi:10.1016/j.healun.2024.02.004)
Supplement: supplemental materials [file NIHMS2026126-supplement-supplemental_materials.docx]

**SUPPLEMENTAL MATERIALS**

**Supplemental Table 1**. Baseline social determinants of health by sex among adult PAH patients enrolled in PHAR

|  | **Total**  **(N = 1,891)** | **Male**  **(N = 466)** | **Female**  **(N = 1,425)** | **Std Diff** |
| --- | --- | --- | --- | --- |
| **Education**, n (%n) | *n = 1,864* | *n = 460* | *n = 1,404* |  |
| No Degree | 136 (7%) | 38 (8%) | 98 (7%) | 0.19 |
| High School Graduate | 1,059 (57%) | 288(63%) | 771 (55%) |  |
| College Graduate | 490 (26%) | 95 (21%) | 395 (28%) |  |
| Post-Graduate Training | 179 (10%) | 39 (8%) | 140 (10%) |  |
| **Income**, n (%n) | *n = 1,411* | *n = 355* | *n = 1,056* |  |
| <$25,000 | 478 (34%) | 121 (34%) | 357 (34%) | 0.05 |
| $25,000 - $74,999 | 520 (37%) | 131 (37%) | 389 (37%) |  |
| $75,000 - $99,999 | 150 (11%) | 34 (10%) | 116 (11%) |  |
| >$100,000 | 263 (19%) | 69 (19%) | 194 (18%) |  |
| **Insurance**, n (%n) | *n = 1,861* | *n = 456* | *n = 1,405* |  |
| Public | 901 (48%) | 251 (55%) | 650 (46%) | 0.18 |
| Private | 925 (50%) | 196 (43%) | 729 (52%) |  |
| No coverage | 35 (2%) | 9 (2%) | 26 (2%) |  |
| **Employment**, n (%N) | *n = 1,839* | *n = 458* | *n = 1,381* |  |
| Student | 21 (1%) | 5 (1%) | 16 (1%) | 0.45 |
| Homemaker | 153 (8%) | 4 (1%) | 149 (11%) |  |
| Employed | 602 (33%) | 155 (34%) | 447 (32%) |  |
| Unemployed | 419 (23%) | 129 (28%) | 290 (21%) |  |
| Retired | 595 (32%) | 154 (34%) | 441 (32%) |  |
| Other | 49 (3%) | 11 (2%) | 38 (3%) |  |
| **Health Related Behaviors** |  |  |  |  |
| Ever smoking, n (%n) (n = 1,879) | 846 (45%) | 273 (59%) | 573 (40%) | 0.38 |
| Current alcohol use, n (%N) (n = 1,879) | 610 (32%) | 171 (37%) | 439 (31%) | 0.15 |
| Ever methamphetamine use, n (%N) (n = 1,884) | 298 (16%) | 123 (27%) | 174 (12%) | 0.36 |

*Std Diff = standardized differences. Ever smoking defined as having smoked at least 100 cigarettes in lifetime.*

**Supplemental Table 2.** Subgroup Analyses in Incident and Idiopathic Patients

|  | **Incident (N = 998)** | | | **Idiopathic (N = 817)** | | |
| --- | --- | --- | --- | --- | --- | --- |
| **Baseline Variables** | **Male**  **(n=252)** | **Female**  **(n=746)** | **P** | **Male**  **(n=193)** | **Female**  **(n=624)** | **P** |
| **Age**, Median (IQR) ^c^ | 58 (45, 69) | 58 (44, 68) | 0.83 | 61 (47, 72) | 56 (41, 68) | 0.01 |
| **Race**, n (%N) ^b^ |  |  |  |  |  |  |
| White | 196 (78%) | 579 (78%) | 0.65 | 155 (80%) | 468 (75%) | 0.30 |
| Black/African American | 26 (10%) | 93 (13%) |  | 18 (9%) | 87 (14%) |  |
| Asian | 7 (3%) | 23 (3%) |  | 8 (4%) | 17 (3%) |  |
| Native American | 3 (1%) | 8 (1%) |  | 2 (1%) | 4 (1%) |  |
| Pacific Islander | 0 (0%) | 2 (<1%) |  | 1 (1%) | 2 (<1%) |  |
| Mixed or Unknown Race | 20 (8%) | 41 (6%) |  | 9 (5%) | 46 (7%) |  |
| **PAH Diagnosis**, n (%N) ^b^ |  |  |  | n/a | n/a | n/a |
| Idiopathic | 106 (42%) | 335 (45%) | <0.01 |  |  |  |
| CTD-associated | 42 (17%) | 280 (38%) |  |  |  |  |
| Drug/toxin-associated | 47 (19%) | 66 (9%) |  |  |  |  |
| Portopulmonary | 35 (14%) | 41 (6%) |  |  |  |  |
| Heritable | 9 (4%) | 21 (3%) |  |  |  |  |
| HIV-related | 13 (5%) | 3 (<1%) |  |  |  |  |
| **Hemodynamic**, Median (IQR) |  |  |  |  |  |  |
| RAP, mmHg | 10 (6, 14) | 9 (5, 14) | 0.15 | 10 (6, 14) | 9 (6, 14) | 0.70 |
| mPA, mmHg | 49 (41, 58) | 48 (39, 57) | 0.18 | 50 (40, 59) | 50 (41, 58) | 0.27 |
| PAWP, mmHg | 11 (8, 14) | 10 (7, 13) | <0.01 | 12 (8, 14) | 11 (7, 14) | 0.04 |
| PVR, WU | 9 (6, 12) | 9 (6, 14) | 0.01 | 8 (6, 11) | 10 (7, 14) | <0.01 |
| PVRi, WU*m^2^ | 18 (13, 24) | 18 (11, 25) | 0.53 | 17 (12, 24) | 18 (12, 26) | 0.35 |
| CO, L/min | 4.4 (3.6, 5.6) | 3.9 (3.1, 4.9) | <0.01 | 4.5 (3.7, 5.5) | 4.0 (3.1, 5.0) | <0.01 |
| CI, L/min/m^2^ | 2.1 (1.7, 2.6) | 2.1 (1.7, 2.7) | 0.98 | 2.1 (1.8, 2.5) | 2.1 (1.7, 2.6) | 0.96 |
| RVSWI, g*m/m^2^/beat | 14 (11, 19) | 14 (10, 17) | 0.09 | 15 (11, 19) | 14 (11, 18) | 0.43 |
| **Number PAH Therapies** |  |  |  |  |  |  |
| 0 – None | 40 (16%) | 115 (15%) | <0.01 | 24 (12%) | 70 (11%) | 0.04 |
| 1 – Mono therapy | 65 (26%) | 198 (27%) |  | 43 (22%) | 138 (22%) |  |
| 2 – Dual therapy | 134 (53%) | 329 (44%) |  | 99 (51%) | 270 (43%) |  |
| 3 – Triple or greater therapy | 13 (5%) | 104 (14%) |  | 27 (14%) | 146 (23%) |  |
| **Functional Parameters** |  |  |  |  |  |  |
| WHO-FC III or IV, n (%N) | 126 (54%) | 430 (61%) | 0.06 | 78 (44%) | 324 (55%) | 0.01 |
| 6MWD, meters, Mean (SD) | 344 (122) | 317 (122) | <0.01 | 355 (148) | 321 (133) | <0.01 |
| % Pred 6MWD, Mean (SD) | 60 (20) | 63 (22) | 0.07 | 62 (23) | 64 (23) | 0.39 |
| **Mortality** | **Male**  **(n = 207)** | **Female**  **(n = 644)** | | **Male**  **(n = 165)** | **Female**  **(n = 540)** | |
| Univariate HR (95% CI) | Ref | 0.61 (0.44 – 0.85) | | Ref | 0.53 (0.36 – 0.77) | |
| Multivariate HR (95% CI) | Ref | 0.53 (0.35 – 0.81) | | Ref | 0.43 (0.26 – 0.70) | |
| Follow-Up [mo], Med. (IQR) | 20 (11, 37) | 24 (12, 43) | | 20 (12, 38) | 24 (13, 42) | |
| Events | 54 | 115 | | 42 | 77 | |

P = P-value. ^a^ = student’s t-test. ^b^= Chi-squared test. ^c^= Rank sum test. HR = Hazard Ratio. IQR = interquartile range. Multivariate HR controls for age, primary PAH diagnosis (for incident group), body mass index, estimated glomerular filtration rate, methamphetamine use, smoking, six-minute walk distance, number of PAH therapies at baseline, mean pulmonary artery pressure, pulmonary vascular resistance, cardiac index, pulmonary arterial wedge pressure

**Supplemental Table 3.** Missing Data

| **Baseline Covariate** | **Number Missing (of total N=1,615)** | **Percent Missing** |
| --- | --- | --- |
| **Body mass index** | 36 | 2% |
| **Estimated glomerular filtration rate** | 38 | 2% |
| **Methamphetamine Use** | 9 | <1% |
| **Smoking** | 22 | 1% |
| **Six minute walk distance** | 103 | 6% |
| **Number of PAH Therapies** | 1 | <1% |
| **Intravenous Prostacyclin Therapy** | 1 | <1% |
| **Mean pulmonary artery pressure** | 39 | 2% |
| **Cardiac index** | 140 | 9% |
| **Pulmonary vascular resistance** | 146 | 9% |
| **Pulmonary artery wedge pressure** | 57 | 4% |
| **Insurance** | 21 | 1% |
| **Occupation** | 44 | 3% |
| **Education** | 19 | 1% |
| **Income** | 378 | 23% |
| **TOTAL** | **1,054** | **4%** |

*If a participant selected “Don’t Know” or “Decline to Answer” on data collected by survey, the variable was considered missing.*

**Supplemental Table 4.** Additional modeling of the conditions under which an unmeasured factor (U) may account for observed sex-based differences in mortality via collider-stratification bias

| **RR for association of female sex with mortality: 1.0** | **M.U** | **PAH.U** | **RR for association of U with PAH in men: 1** | | | | | |  | **RR _Model_ < RR _Observed_**  Modeling estimates female sex is more protective than the observed data demonstrate (RR _Model_ < 0.60; below the lower limit of the 95% confidence interval of observed data) |
| --- | --- | --- | --- | --- | --- | --- | --- | --- | --- | --- |
|  |  |  | **Prevalence of U in population without PAH** | | | | | |  |  |
|  |  |  | **0.05** | **0.10** | **0.20** | **0.30** | **0.40** | **0.50** |  |  |
|  | **1** | **0.67** | 1.00 | 1.00 | 1.00 | 1.00 | 1.00 | 1.00 |  |  |
|  | **2** | **0.67** | 0.98 | 0.97 | 0.95 | 0.94 | 0.93 | 0.93 |  | **RR _Model_ = RR _Observed_**  Modeling estimates female sex is as protective as the observed data demonstrate (RR _Model_ 0.60 – 0.92; within the 95% confidence interval of observed data) |
|  | **5** | **0.67** | 0.95 | 0.91 | 0.87 | 0.86 | 0.86 | 0.87 |  |  |
|  | **1** | **1.00** | 1.00 | 1.00 | 1.00 | 1.00 | 1.00 | 1.00 |  |  |
|  | **2** | **1.00** | 1.00 | 1.00 | 1.00 | 1.00 | 1.00 | 1.00 |  |  |
|  | **5** | **1.00** | 1.00 | 1.00 | 1.00 | 1.00 | 1.00 | 1.00 |  | **RR _Model_ > RR _Observed_**  Modeling estimates female sex is less protective than the observed data demonstrate (RR _Model_ > 0.92; above the upper limit of the 95% confidence interval of observed data) |
|  | **1** | **1.50** | 1.00 | 1.00 | 1.00 | 1.00 | 1.00 | 1.00 |  |  |
|  | **2** | **1.50** | 1.02 | 1.04 | 1.06 | 1.07 | 1.07 | 1.07 |  |  |
|  | **5** | **1.50** | 1.08 | 1.12 | 1.16 | 1.17 | 1.15 | 1.13 |  |  |

M.U. = risk ratio for association of U with mortality. PAH.U = risk ratio for association of U with PAH among women. Assumptions: 1) prevalence of PAH among men = 5 cases per million^44^; 2) Risk ratio (RR) for effect of female sex on PAH = 3.0 (as seen in PHAR); 3) mortality of men versus women in the general population = RR 1.0 (equivalent); 4) RR for the association of U with mortality = varied between 1.0 and 5.0; 5) RR for the association of U with PAH among women = varied between 0.67 and 1.50; 6) prevalence of U in the population without PAH = varied between 0.05 and 0.50. Table is color-coded according to whether modeling of collider-stratification bias predicted RR which were below (red), equal to (green), or above (blue) the 95% confidence interval for the observed unadjusted RR for mortality by sex in PHAR (RR 0.74; 95% CI 0.60 – 0.92).

**Supplemental Figure 1.** Sensitivity Analysis with Multiple Imputation of Missing Data: hazard ratio and 95% confidence intervals for unadjusted and adjusted mediation models of the association between sex and mortality

*
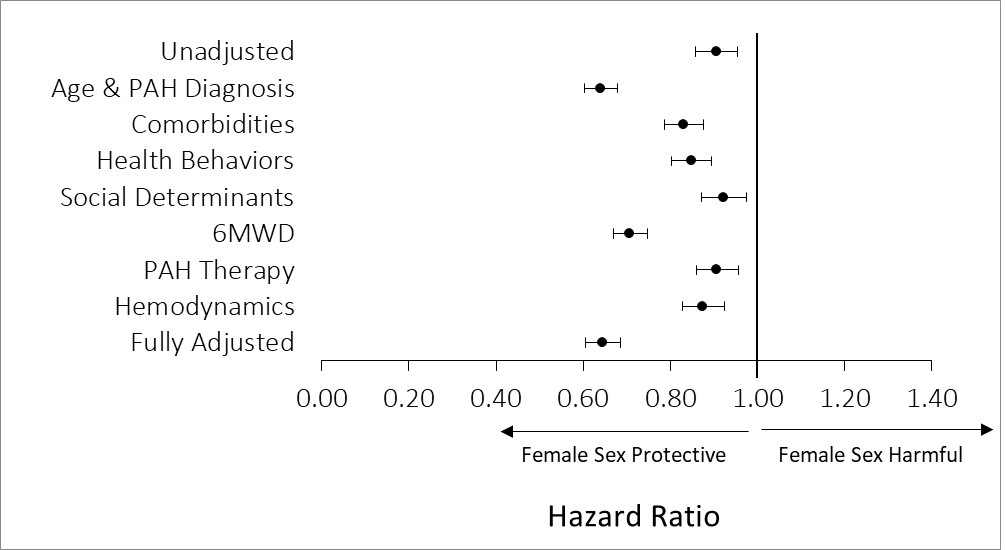
*
